# A qualitative study on the implementation of a holistic care package for control and management of lymphoedema: experience from a pilot intervention in northern Ethiopia

**DOI:** 10.1186/s12913-021-07088-7

**Published:** 2021-10-08

**Authors:** Oumer Ali, Mersha Kinfe, Maya Semrau, Abebayehu Tora, Abraham Tesfaye, Asrat Mengiste, Gail Davey, Abebaw Fekadu

**Affiliations:** 1 CDT-Africa, College of Health Sciences, Addis Ababa University, Addis Ababa, Ethiopia; 2 Centre for Global Health Research, Brighton and Sussex Medical School, Falmer Campus, University of Sussex, Brighton, BN1 9PX UK; 3 Department of Sociology, Wolaita Sodo University, Sodo, Ethiopia; 4 College of Health Sciences, School of Public Health, Addis Ababa University, Addis Ababa, Ethiopia

**Keywords:** NTDs, Lymphoedema, Disability, Psychosocial

## Abstract

**Background:**

Neglected Tropical Diseases (NTDs) such as podoconiosis, lymphatic filariasis (LF) and leprosy mainly affect communities in low resource settings. These diseases are associated with physical disability due to lymphoedema as well as poor mental health and psychosocial outcomes. Integration of care across these NTDs at primary health care level, which includes mental health and psychosocial care alongside physical health care, is increasingly recommended.

**Methods:**

A holistic integrated care package was developed and piloted as part of the EnDPoINT project in Gusha district, Awi zone, Ethiopia. The intervention was conducted at the health care organization, health facility and community levels. To assess the impact of the care package in terms of acceptability, scalability, sustainability and barriers to implementation, a qualitative study was conducted in January 2020. This included four focus group discussions (29 participants) and ten key informant interviews with decision makers, health professionals, patients, and community representatives.

**Results:**

The integrated lymphoedema care package was found to be efficient compared to vertical programs in saving time and resources. It also resulted in improved awareness of the causes, treatment and prevention of lymphoedema**,** in marked improvements in the lymphoedema, and in reduced stigma and discrimination. The care package was found to be acceptable to patients, health professionals and decision makers. The barriers to integrated care were unrealistic patient expectations, inadequate dissemination across health workers, and poor transportation access. Health professionals, decision makers and patients believed the integrated lymphoedema care package to be scalable and sustainable.

**Conclusion:**

The integrated holistic care package was found to be acceptable to patients, health professionals and decision makers. We recommend its scale-up to other endemic districts.

**Supplementary Information:**

The online version contains supplementary material available at 10.1186/s12913-021-07088-7.

## Background

Neglected Tropical Diseases (NTDs) are diverse in biological and transmission characteristics; they predominantly affect populations in low and middle-income countries in sub-Saharan Africa, Asia and Latin America. NTDs predispose to long term disability and poverty [1]. Lymphoedema is a chronic condition which affects the lymphatic system, and is manifested by swelling of the body tissues, most commonly the arms and legs. In tropical countries, most cases of lymphoedema are attributable to lymphatic filariasis (LF) and podoconiosis, and a smaller proportion to leprosy [2]; all of these are NTDs. The WHO-recommended basic lymphoedema management activities include limb washing, elevation, exercise, skincare, wound-care (applying creams and dressings) and the protection of feet with appropriate footwear [3].

In Ethiopia, in 2015, there were about 1.5 million cases of podoconiosis, and the disease was endemic in 345 districts of the 775 districts surveyed. In the same calendar year, nearly 36 million people were living in areas with podoconiosis prevalence exceeding 1% [4]. In the same survey, 75 of 658 surveyed districts were found to be endemic for LF. Including the 37 previously studied districts, a total of 112 endemic districts in Ethiopia (or nearly 12 million people) were at risk of LF [5]. In Ethiopia, in 2015, a total of 3970 new leprosy cases were registered. The proportion of children among new cases of leprosy was 14.2%, females were 31%, [6] and 10.6% of new cases of leprosy had grade II disability at diagnosis during the same calendar year [6]. The overall prevalence of lower limb lymphoedema in Ethiopia was 6.2% (95% confidence interval [CI] 6.1–6.4%) [7]. Nationwide mapping demonstrated that podoconiosis accounts for approximately 64.8%, LF for 13.2% and leprosy for 12.8% of the total burden of lymphoedema in Ethiopia [7]. The lymphoedema prevalence rate in Amhara region where the study was conducted is 62.7 per 10,000 population [8].

The integrated delivery of community-based interventions for helminthic NTDs in co-infected communities has been shown to result in reduced prevalence of NTDs and more effective control with greater coverage compared to routine vertical delivery [9]. Moreover, integrated interventions have been found to be more feasible and cost effective than vertical care [9]. Integrated community-based interventions for non-helminthic NTDs have also been shown to lead to a reduction in incidence and burden of these NTDs, and to result in extended coverage and sustained community acceptance [10]. Similarly, integrated interventions for skin NTDs have been found to be effective and efficient in relation to reduction of NTD-related morbidity, alleviation of poverty [11], and have resulted in capacity building, awareness creation and motivation of health workers. Conversely, integrated care may have some limitations, including loss of specialized expertise due to loss of vertical care, lack of adequate trained staff, and staff turnover following training [11].

Skin NTDs such as podoconiosis, lymphatic filariasis (LF) and leprosy mainly affect communities in low resource settings, and are associated with physical disability due to lymphoedema as well as poor mental health and psychosocial outcomes. Systematic reviews [12–14] have shown that such stigmatized chronic NTDs are associated with comorbid mental health conditions more than other chronic diseases [15]. Integration of care which includes mental health and psychosocial care alongside physical health care, across these NTDs at primary health care level, is therefore increasingly recommended.

The burden of lymphoedema includes health-related, psychosocial and economic burdens, stigma and discrimination. One of the health effects is reduced mobility related to limb swelling. In addition, lymphoedema leads to acute attacks [16, 17] which are manifested by symptoms of inflammation including severe pain, rigours and chills. In addition to these physical health outcomes, patients face considerable psychosocial burdens. Due to exclusion from social activities and decision-making roles, there is a significant impact on social, psychological and mental health outcomes including poor quality of life [18–20], mental distress [21] and depression [22, 23]. Lymphoedema also leads to disability so that affected individuals are unable to work, which may result in a reduction in productivity [24] that affects the family, local, regional, and even national economy. Finally, patients face stigma and discrimination [25, 26] by the community, and may also stigmatise themselves.

In Ethiopia, whilst leprosy has been managed under the TB-Leprosy program and so treatment has been provided through the TB program, to date, care of podoconiosis and LF lymphoedema has received little attention from the government system.

The Excellence in Disability Prevention Integrated across Neglected Tropical Diseases (EnDPoINT) mixed-methods research project aims to integrate holistic care for podoconiosis, LF and leprosy into primary health care facilities in Ethiopia. The project is being implemented in three phases. In Phase 1, a holistic integrated care package involving physical health, mental health and psychosocial care components was developed for the three diseases. In Phase 2, the care package was piloted in Gusha cluster of Guagsa Shikudad district, Awi zone. In Phase 3, it is being implemented and scaled-up in three districts in Awi zone. The detailed protocol of the EnDPoINT project has been published elsewhere [27]. This paper relates to qualitative work conducted during Phase 2 of EnDPoINT.

The aim of this study as part of the EnDPoINT project were: 1) to assess implementation fidelity by determining the integrated care package’s acceptability, scalability, sustainability and the barriers to its implementation; 2) to assess specific program outcomes, including reduction of misconceptions, reduction of stigma and improvement of clinical outcomes.

## Methods

### Setting

The study was conducted within the Awi zone of Ethiopia, which is divided into seven districts (or *woredas*), and is one of ten zones in the Amhara Region in the North-West of Ethiopia. According to the Ethiopian Census, Awi zone had a population of 982,942 in 2007 (with each district having populations of between 8000 and 31,500); the current population is projected to be around 1.2 million. In the zone 87.5% of the population live in rural areas and 12.5% in urban areas. Injebara is the administrative centre. On average, there were 4.6 people living in each household in Awi zone in 2007. The majority of the population are from the Age-Awi (59.8%) and Amhara (38.4%) ethnic groups, and their first language is primarily Amarigna (53.4%) and Agew-Awinigigna (45.0%), with over 60 other ethnic groups and languages spoken as first language. The majority of the population are Ethiopian Orthodox Christians (94.4%), with a minority being Muslim (4.5%) or of other religions [28].

### Description of the care package

The integrated, holistic care package was developed in Phase 1 of EnDPoINT based on a situational analysis, a literature review, Theory of Change workshops, and qualitative studies. The care package includes interventions that are conducted at three levels of the health system: health care organization, health facility and community level. Components of the intervention are program management, community engagement, awareness raising, stigma reduction, case finding, assessment, diagnosis, treatment services, and patient counselling.

A major part of the intervention was training of trainers (TOT) on integrated management of podoconiosis, LF, and leprosy, and on the management of common mental disorders. The training had both theoretical and hands-on components.

**Fig. 1:**
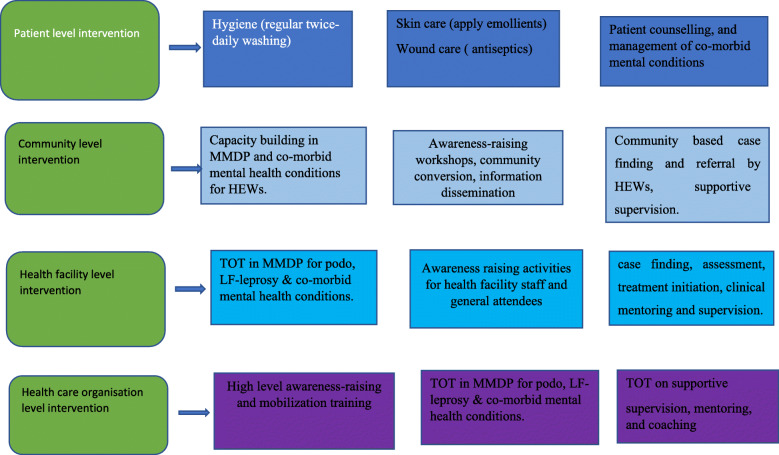
MMDP and co-morbid mental conditions interventions at different levels of care

In order to alleviate discrimination and mis-information, the care package includes awareness-raising activities such as awareness-raising among general attendees at the health center, community awareness-raising workshops, community conversation, and information dissemination to the wider community. As part of monitoring and evaluation, continuous supportive supervision was conducted to ensure sustainable integration of the care package into the Primary Care Unit. The intervention care package is depicted in Fig. 1(MMDP and co-morbid mental conditions interventions at different levels of care. The details has been described in [29]).

### Study design

During Phase 2 of EnDPoINT, to which this study relates, the care package was piloted in Gusha cluster of Guagsa Shikudad district in Awi zone. Alongside quantitative evaluations (which will be published separately), qualitative methods were used to collect data through key informant interviews (KIIs) and focus group discussions (FGDs). Sample size was determined by a data saturation approach, that is, data collection was continued until either sufficient information had been obtained or further data collection failed to generate additional themes. The more information the sample holds, with respect to the aim of the study, the lower the sample size required [30, 31].

We used purposive sampling techniques - members of the EnDPoINT research team contacted key stakeholders and asked them to participate in the FGDs or KIIs.

### Data collection procedures

We conducted four FGDs with health professionals, decision makers, community representatives and patients, and ten KIIs with the head and vice head of the District Health Office, the District NTD focal person, the District leprosy focal person, the head of the Health Center, the NTD focal person for the Health Center, patients, and health extension workers (HEWs). All participants who were approached consented to participate.

The qualitative data collection was conducted in January 2020. A semi structured interview guide was used. Interviews were conducted in the local language, Amharic. OA, MK and AM conducted the interviews. All participants were encouraged to contribute and be heard. Patients and non-patients participated in separate focus groups. There were six to eight participants per group, and the duration of the discussion was between 40 min and 1 h.

We also conducted 10 KIIs with Head of zonal and district health office, NTD focal person, TB (tuberculosis) and Leprosy focal person, Head of health centre, Health professionals and HEWs (Health Extension Workers). The KIIs lasted between 30 and 40 min each. We made sure that the interviews were conducted at places chosen by the participants. Prior to the interviews, we spent time chatting with participants to make them feel comfortable with the interview process and to build rapport. During the interviews, we engaged with participants sensitively, with empathy and care.

### Analysis

All interviews were transcribed verbatim and translated into English by researchers from Addis Ababa University who are bilingual in Amharic and English. The audio-recorded interviews were transcribed and translated into English before coding. The accuracy of all transcripts was checked by comparing them with the audio-recorded interviews. The analysis was conducted in English. Coding was done using NVivo 12 plus by OA and MK and was further reviewed by the research team to ensure consistency with the codes and data. Thematic analysis was used, starting with predefined themes in the interview guides, and open and axial coding was followed to identify new themes emerging from the data. Pre-determined themes were based on the guiding questions developed following the three Theory of Change (TOC) workshops during Phase 1. A conceptual framework was developed following the TOC workshops; the main components of the framework were program resources, capacity building, case identification, service delivery, long-term outcome and impact of the intervention.

### Ethical considerations

Ethical approval for the study was obtained from the Brighton and Sussex Medical School Research Governance and Ethics Committee in the UK (9th November 2018, Ref Number ER/BSMS9D79/2), and the Institutional Review Board of the College of Health Sciences at Addis Ababa University in Ethiopia (26th September 2018, Ref Number 061/18/CDT).

## Results

Among the 39 study participants, 29 were male and 10 were female; the age range was 24 to 62 years. Among these, six were single and 33 were married. They belong to varied professional categories: five Public health experts (MPH), seven health officers (BSc), six clinical nurses (BSc), two public health nurses (diploma), one druggist (diploma), two Health Extension Workers (HEWs), and sixteen farmers. All except one (who was Muslim) belonged to the Orthodox Christian religious group. Ten people participated in the KIIs and 29 participated in the FGDs.

A range of themes and sub-themes were identified. Themes included burden of lymphoedema on affected individuals, misconceptions about NTDs, stigma and its mitigation, advantages of integrated care for NTDs, acceptability of the care package, outcomes of treatment, scalability and sustainability of care, while sub-themes included integration of care across NTDs, psychosocial care integrated with limb care, barriers and solutions to implementing the integrated care package, acceptability by patients and caregivers, acceptability by health care workers and acceptability by decision makers. The predetermined themes included stigma and its mitigation, advantages of integrated care for NTDs, acceptability of the care package, outcomes of treatment, and scalability of care. The themes that emerged during the analysis included burden of lymphoedema on affected individuals, misconceptions about NTDs, and sustainability of the care package.

### Burden of lymphoedema on affected individuals

#### Health burden

Podoconiosis, LF and leprosy can all lead to lymphoedema complicated by acute attacks, which are characterised by severe pain, fever, chills and difficulty walking.

Before starting the treatment I felt chills and rigor whenever I returned from work. I couldn’t even eat food because of the pain. I take a cup of coffee but am still in pain. That is the acute attack, which causes severe damage to our health and effectiveness in work [FGD Participant, 47 year old female patient]

The last two years were especially difficult for me; it gets difficult even to get out of my house. My family worried about me, others blame me that I fell while working. I feel weak. [FGD participant, 49 year old male patient]

#### Psychosocial burden

NTDs not only have devastating physical outcomes, but also tremendous social, psychological and mental health outcomes. Undermining personal dignity and exclusion from decision making roles and social activities were common experiences of lymphoedema-affected individuals. These experiences had a negative impact on the mental health of lymphoedema patients. As some participants stated,

In our workplace our work-mates used to belittle us due to our condition. They didn’t consider us equal for social or political positions, which is painful. [FGD participant, 58 year old female decision maker (DM)]

People with this problem definitely have mental health problems or these might happen in their lifetime. These mental health problems are neglected as there is not even one psychiatric professional in the health centre.’ [Decision maker, 31-year-old male]

#### Economic burden

Participants also reported that NTDs have a significant impact on the economy of the family and there is a possibility that more than one person in a family is affected by these diseases.

In a single family there may be three or four affected individuals. The affected individuals are poor as they can’t work due to disability. Even if they have farmland, they can’t cultivate it, rather they go begging. [FGD participant, 50 year old female community representative (CR)]

#### Burden due to stigma and discrimination

The community tend to stigmatize and discriminate against individuals affected by lymphoedema, a highly visible condition. They avoid marriage to individuals affected by lymphoedema, as they believe the disease will pass to their offspring.

There were a husband and wife with huge leg swelling in the neighbourhood, two years back she prepared ‘*Tela*’ (a local alcoholic beverage) and took it to the church to celebrate a holiday, but people refused to drink what she had brought. [FGD participant, 47 year old male patient]

Even in the case of marriage proposal, if the man has a leg problem, the woman’s family do not allow their daughter to go to his family. They think that either the disease will be transmitted directly to their daughter or passed genetically to their grandchild. [FGD participant, 48 year old female CR]

The stigmatization is not only from the unaffected community but also from the patients themselves.

The problem is not only from the other side, patients also isolate themselves fearing that the bad smell would disturb other people, so it is a two-way problem. [FGD participant, 28 year old male Health Professional (HP)]

#### Misconceptions about podoconiosis, LF and leprosy

For podoconiosis, most of the patients participating in the interviews thought that is was caused by a curse. Few mentioned barefoot contact with the soil for a long period of time as a cause.

People believe that podoconiosis is caused by a curse from God - even though they can afford to buy shoes, they don’t want to buy and wear them. However, had they known that walking bare footed is the cause of this illness, they will prioritize shoes even above food and drink. [FGD participant, 51 year old female CR]

Be it podoconiosis, LF or leprosy, most people did not understand that it could be prevented or treated medically. Similar to podoconiosis, they believed that LF and leprosy were caused by a curse from God. Most did not believe that leprosy is caused by a bacteria and LF by a parasite

Previously, there was no awareness about the disease; they considered themselves inferior to everybody and they thought the diseases had no cure but now we follow them and they know that if they preserve their hygiene there is a cure and it doesn’t transmit through blood lines. [HEW, 28-year-old female]

Some people think that we brought this on ourselves as we did something wrong that God didn’t like. It is a matter of education: those who are illiterate think like that, but the ones with some knowhow show us empathy and support in everything. [FGD participant, 47 year old male patient]

The misconceptions around podoconiosis, LF and leprosy are not limited to patients - there have been reports of misconceptions even among health professionals regarding the treatment of the disease [32]. NTDs, especially podoconiosis, are inadequately addressed in health professionals’ training curricula.

I am a focal person at the health center. First, when I came to this profession, to tell you the truth, I was treating only other cases, and also the acute attack cases came due to this problem. We didn’t understand their [patients with podoconiosis] problem, despite providing pain relief for them. In addition, there was nothing that we got from the curriculum too. We didn’t know whether they will get improvement by foot hygiene. But at this time, after attending the training, first we know about it and we also create awareness in the community too. So, I believe that we will solve the problem. [Decision maker, 28 year old male]

‘I was shocked when I heard that there are about 251 cases [of lymphoedema] at Gugsa cluster. I mean, sometimes we do see those cases even though we don’t differentiate specifically whether it is podo or Lymphatic filariasis as both of them have similar differential diagnoses, we may not specifically know the case. Moreover, I may not have the reference book at the table, so, that means I may mis-diagnose the case at that time. Therefore, it is necessary to have an orientation. [FGD participant, 35 year old male HP]

### Integration of the holistic care package into the primary health system

#### Perception of decision makers about integration of lymphedema care

To operationalise the EnDPoINT project, advocacy activities were started at the leadership level. Training on integrated care of the three NTDs and psychosocial care was provided to health professionals. Then awareness-raising workshops were conducted in health facilities. Community awareness was addressed through facilitated community conversations.

It is good to do things in an integrated way as you can save more time and resources. Therefore, integrating the care package of the three diseases in Gusha health centre proves we can achieve that. It saves much needed time and money, the pilot project shows we achieve the three cares in one. As I am a leprosy officer I gained good experience, got a lot knowledge that I didn’t have before. Therefore, the integration of treatment package was good and effective which needs to continue. [ Decision maker, 37 year old male]

Each of the diseases is currently included in the check list and I can tell you that they are being supported and the service is being provided. Even though these diseases were neglected during the previous times, at present, they are being included in the program. Hence, I think it will be strengthened more in the future and we will work more by collaborating with partner organizations to make the program more successful and to make sure the community benefits from it. [Decision maker, 42 year old male]

#### Perception of health professionals about integration of mental health care

Patients with lymphoedema stigmatise themselves and feel inferior compared to their healthy counterparts. Due to this, they are at risk of mental distress. Thus, integration of lymphoedema care with psychosocial-mental health care is very important.

Patients live stressed life and I don’t think that they feel good internally because they think they are discriminated against by others or may think they are inferior to others because they aren’t able to do what others can do for living, so including a psychological intervention benefits them more. [FGD participant, 62 year old male DM]

Considering the local health condition, previously mental health services were given only at hospital level, not at health centre level. However, through the CDT-Africa/EnDPoINT project mental health training has been given to health centre-level healthcare professionals. A three-day theoretical training and five-day practical training was delivered.

Mental health services were not being provided in health centres, it was totally an ignored work. [FGD participant, 55 year old male DM]

Mental health problems are neglected, there is not even one psychiatric professional at the health centre. At *woreda* level, training to give an integrated service was given to health professionals working in the health centres. This training was given for those who work on morbidity management to support the psychosocial-mental health care. Therefore, I believe the psychosocial work is a vital part of the treatment package and goes hand-in-hand with the treatment and prevention work. [Decision maker, 31 year old male]

### Perceived barriers to integration of the holistic care package

#### Unrealistic expectations

One of the barriers to implementing integrated care comes from the patients themselves. After providing the health education and delivering start-up supplies to practice self-treatment, it is expected that patients continue to receive the services covering the costs necessary. However, they expect the cost of supplies to be covered by the government.

There are a lot of patients who are currently enrolled in the service and there are patients who got help before. Several patients want everything to be covered by the government, they want the government to give them all services for free. Maybe this will be a challenge. [Health professional, 30 year old male]

#### Inadequate dissemination across health workers

Another barrier is lack of cooperation from officers and health workers at the health center, as they consider the work to be only for the trained focal persons and heads.

There is a low level of awareness among health workers, except those who attended the training, since health professionals didn’t learn much about podoconiosis or LF from their college time. [Decision maker, 37 year old male]

#### Poor transport access

A further challenge is lack of transport. Either patients are poor and cannot afford transport to the health centre where the integrated lymphoedema care is given, or are too disabled to walk because of the leg swelling.

While they want to come here, they can’t find transport and may spend the night on the street. Since these patients are poor, they don’t have money for a ‘bajaj’ [A form of taxi with three wheels that can carry up to five passengers]. So my first request is to bring the service to health post level. [FGD participant, 57 year old male CR]

### Acceptability of care package

#### Perception of patients

Patients were very comfortable with the integrated care package. They described how much relief the self-treatment brought. They acknowledged the government and the project for reaching them with such effective measures. They asked for more education and that the counselling services be continued. They were ready to purchase the materials necessary for long term management themselves.

First, the package has much more acceptance after patients have gone through the agony of suffering and see that it is better and more comfortable. They are accepting it very well and they are also strengthening the association by contributing money. [The package] is accepted by both the patients and their caregivers. [HEW, 26 year old female]

The patients say ‘GOD came to us, we were created as human but were not living like humans, but now we have become human again”. For your information the names of our self-help groups are very unique. For example, one of the name is “Fetari Dereselign” which means “the almighty God reached for us”, there is no satisfaction like this. [Decision maker, 31 year old male]

#### Perception of health care workers

Capacity building activities were delivered to health professionals, health extension workers and district officers. Following this, there was tremendous motivation in the health sector to implement the integrated lymphoedema package, and the providers gave special priority to the program. The health professionals were very happy about integrated care and they said that there had never been a job that gave them more satisfaction than this one.

The providers are delivering compassionate and respectful care. When demonstrating foot care they kneel and wash the feet. Previously they may have cleaned and cared for wounds, but not washed a patient’s feet. This project teaches us to be more humble and compassionate to our patients, to tackle problems more thoroughly. It is good to do things in an integrated way as you can save more time and resources. The integration of the three diseases gives us great experience for other diseases. [Decision maker, 37 year old male]

Now every patient you get will bless you. Every one we find is blessing us. This will push you to do more work. You will never have a reason to stop working while they are blessing you for what you did. So the health professionals are committed. The health professionals at the health centre will go to each *kebele* [The smallest administrative unit consisting about 1000 head of households] and observe patients’ progress covering their own transport cost. [Decision maker, 31year old male]

#### Perception of decision makers

Decision makers reported how impressed they were with the levels of collaboration seen in the community around the integrated lymphoedema package. They saw the introduction of the package as an opportunity for local government to get behind services clearly appreciated by the community.

I have been noticing the effectiveness of integration. After project initiation, all responsible bodies from zone to *kebele* level have been actively participating and helping us. When we go out to *kebele* level to conduct supportive supervision, [community] leaders and health extension workers are doing a great job in collaboration. [FGD participant, 28 year old male HP]

This is a condition that the government itself has previously neglected. But now attention is given, and using this attention as a good opportunity, the management is committed to make the service available and to help the community address the situation. [Decision maker, 31 year old male]

### Outcomes of the holistic care package

#### Improved awareness about the cause, treatment and prevention of lymphoedema

Respondents noted increased understanding of lymphoedema and strategies to prevent it compared with the pre-intervention package situation.

Now people understand the cause and that it is possible to treat the disease. Everyone is urging the community not to go barefoot and to keep their feet clean. Even in the farming areas, they wear plastic boots. [FGD participant, 48 year old male patient]

Healthcare workers facilitated the establishment of self-help groups. These groups contain eight to twelve patients, and have a chairperson, secretary and audit officer. Each member contributes a specified amount of money each month. The main role of the self-help group is health education including disease prevention, health promotion, and stigma reduction.

I participated in the training for trainers of self-help groups. After my training, I have been closely following the members of our association. We use the association to educate each other and follow our progress. Following our education, we have been practicing washing our feet and skin care, which has brought many changes. People who have sleepless nights before are now enjoying their times peacefully. [FGD participant, 47 year old male patient]

#### Improved lymphoedema condition

Proper continuation of lymphoedema care resulted in considerable reduction of pain, of frequency of acute attacks and of the extent of swelling.

By now there is no problem at all. I was in pain all days of the month before I started this treatment, but now I haven’t had a single day of illness after following the advice from health workers. I follow every procedure as recommended and that makes everything well. Now I can wear size 40 shoes, while previously I couldn’t even wear size 43 shoes. [FGD participant, 47 year old male patient]

Continued lymphoedema care also resulted in increased productivity and improved quality of life.

After I started this treatment, thank God, I am in peace. Unfortunately, our fathers didn’t get this opportunity. Now I am farming equally as my friends do. Thank you so much, you help us a lot and [the package] improves our quality of life. [FGD participant, 49 year old male patient]

Some could barely move outside their home before starting treatment, but now they go out and do their business just like a normal person. Their feeling of shame about participating in social occasions because of the smell has gone. [Decision maker, 37 year old male]

#### Reduced stigma and discrimination

Patients and community members described changes in levels of stigma and discrimination they faced following implementation of the integrated care package.

Previously, people had a tendency not to eat the food we prepared. Now this education and community conversation comes. We use our knowledge to convince them, and some are changed for the good. [FGD participant, 47 year old male patient]

My sister used to prepare food to be served in the church but while the men ate it, the women did not. That hurt my feelings deeply. Now we’ve started this treatment and help each other with the self-help group, we wear our shoes and go out as equals to anyone around. My sister is following treatment closely and by now she is in a near normal condition. Recently in church, I have witnessed the girls who used to refuse the food take it as normal, and I feel happy to see that. [FGD participant, 53 year old male patient]

#### Perception on scalability of care

Patients, healthcare workers and decision makers all considered the integrated package to be successful and urged its wider introduction. Patients pointed to wider relief of suffering and returning more people to full lives in society, while decision makers stressed the importance they placed in the scientific rigour with which the package had been implemented and evaluated.

It would be good if this organization could work in other districts. While working on these activities, I believe that the health office will take the initiative to work in other areas which have similar problems. So, it will be very important for the community if you scale up the program to other districts so as to help the community to get rid of this problem and have healthy and productive citizens. [FGD participant, 52 year old male DM]

For the sake of people, if you continue the service in other parts of the country in which this problem exists, I am very happy. Because of the modality of the treatment, we are happy. [Health professional, 28 year old male]

We appreciate the follow up you are conducting on the progress of the patients. You are doing it in scientific manner, which is nice. You are helping the patients and relieving many sufferings. We even expect the ministry of health to take this idea and scale it up nationwide. [Decision maker, 32 year old male]

#### Perception on sustainability of care

One of the activities intended to ensure sustainability of the package was the training of trainers (TOT) for health professionals. Those who took part in the TOT were expected to cascade training to the remaining health care workers. This aims to protect against high turnover among healthcare workers, which has been a problem for the sustainability of other new healthcare interventions.

It is good to see as many health workers as possible trained in this to ensure the sustainability of the work after the project phases out. For us we will try to use every level of government to sustain it. [FGD participant, 57 year old male CR]

Another factor that might influence sustainability is the ability and motivation of patients to continue buying the simple consumables needed (for example, soap and ointment). Most of the supplies for hygiene-based lymphoedema management are easily accessible, for those with extensive swelling, shoes of an appropriate size are not available in any local shops.

The community has the awareness and they can even purchase the materials by themselves. Previously it was the organization that provides the materials. And they [patients] are so happy with the change they witnessed and consider it sustainable. [Health professional, 28 year old male]

We can find soap, Vaseline and other materials in the local shops, but shoes of our size are difficult to find, so that is the main problem to be addressed by the government. [FGD participant, 53 year old male patient]

Integrating lymphoedema care into the existing Health Extension Program (HEP) is another factor vital to the sustainability of the intervention. Decision makers suggested that since cost is one of the challenges for sustainability, incorporating the intervention into the HEP might reduce the long-term cost.

The government is trying to accommodate specific programs into the Health Extension Program. For example, personal hygiene is among the packages of the HEP, so we can take podo, LF, leprosy, skin care and washing practice and then contextualize them with the existing Health Extension Programs. The HEP is one of the most sustainable programs the government has, so we can use it to solve both the sustainability and budget issues. There are some efforts to include podoconiosis care in it. [Decision maker, 32 year old male]

## Discussion

In this study we have observed misconceptions about the cause, prevention and treatment of lymphoedema due to podoconiosis, LF and leprosy. In regard to podoconiosis, the affected community believes that it is caused by a curse; although some mention that it is hereditary, most do not realise that it is caused by long term barefoot exposure to red clay soil. Podoconiosis is caused by both genetic and environmental factors [33, 34]. Similarly, a common misconception is that LF and leprosy are caused by a curse, when LF is actually caused by the parasites *Wuchereria bancrofti* and *Brugia spp* and transmitted by mosquitos, whereas leprosy is caused by *Mycobacterium leprae*.

Integrated lymphoedema care is a prefered modality of care among our study participants belonging to decission makers and health professionals. Similar to our findings, integrated NTD care was found feasible and cost effective in reduction of morbidity [9, 11]. Integrated care on NTDs was also resulted in reduction of disease burden [9, 10], and increased coverage [9, 10]. In contrast to our findings, integrated care has its own negative effects as it lead to loss of specialised proficiency and a tendency for lack of adequate trained staff [11]. As lymphoedema due to NTDs results in poor psychosocial and mental health outcomes, integration of lymphoedema care with mental health care at primary health care units is vital [35].

The integrated holistic care package was found to be acceptable by patients. They reported feeling comfortable with the treatment package and witnessed significant improvements associated with their illness. They were happy with the health education and the counselling activities. Though most were ready to purchase the necessary supplies for the long term self-care, a few of them wanted the government to give them the supplies for free. Similar to patients, the health professionals found the care package to be acceptable. The gratitude from patients enhanced the motivation of health professionals and they claimed that they had never had any job which gave them greater satisfaction than the integrated holistic care package. Unlike previously, when mental health care for people affected by lymphoedema was neglected, in this study, integrated NTD-mental health care won the support of decision makers, who believed that it made efficient use of the work force.

The outcomes of the integrated holistic care package included improved awareness about the causes, treatment and prevention of lymphoedema**;** improved lymphoedema condition, and reduced stigma and discrimination. Post-intervention, there was enhanced understanding about ways of controlling and preventing lymphoedema. A self-help group was established, leading to increased information sharing and persistent self-care. Appropriate lymphoedema care was followed by reduction of pain [36], decreased frequency of acute attacks [17, 36, 37] and profound reduction of swelling [38]. In addition, it resulted in enhanced productivity and improved quality of life [38]. Post-intervention, patients and community members noticed a reduction in stigma and discrimination.

Though the integrated holistic care package was reported to have advantages by participants, it was also found to have some drawbacks. These include unrealistic expectations by patients, inadequate cooperation of health workers, and poor transportation access. Patients expected the government to cover the cost of the supplies required for integrated lymphoedema care. There was a tendency to push integrated lymphoedema-mental health care activities towards those healthcare professionals who had attended TOTs. As part of the solution, orientation of a wider range of health workers may help develop capacity more sustainably. Finally, patients may have poor transport access to the health centre where the integrated care is available, either because they are poor and cannot afford transportation or because the lymphoedema leads to reduced mobility. Thus, delivering this service at the health post level, nearer to the community, is proposed.

Health professionals, decision makers and patients strongly supported scale-up of the integrated package to the remaining endemic districts in the zone. They even expected the Ministry of Health to scale it up nation-wide. One of the justifications for scalability is the scientific soundness of the implementation and evaluation mechanisms. Another important justification for scale-up is the relief felt by patients that, as their symptoms reduced, they were able to return to their duties and could once more become productive citizens.

The integrated care program is potentially sustainable. Factors which contribute to sustainability include the training of trainers (TOT) given to health professionals, the ability of patients to buy the consumables and the potential for integration into the existing health extension program. The TOT provided to the health professionals enabled them to cascade this training to the remaining health professionals in the health facility. This helps to prevent loss of expertise secondary to staff turnover, which often acts as a barrier to the sustainability of newly initiated programs.

Since cost is often a barrier to sustainability, one of the decision makers’ recommendations was incorporation of the integrated care package into the existing Health Extension Program (HEP). The HEP is a highly sustainable government program, and lymphoedema care was thought to fit well into it. Another important issue is ability to buy the essential supplies. By the end of the intervention, patients had the awareness, motivation and ability to buy most of these supplies. The only limiting factor mentioned was availability of shoes appropriate for those with very large swelling, as these were not usually available in the local market.

## Conclusion

The integrated lymphoedema care package supported lymphoedema awareness creation, reduction of stigma and discrimination, and marked improvement in lymphoedema. The care package was found to be acceptable to patients, health professionals and decision makers. We recommend its scale-up to other endemic districts in Ethiopia, and potentially other countries.

## Supplementary Information

**Additional file 1.** Topic guide questions english version.

## Data Availability

The datasets used and/or analysed during the current study are available from the corresponding author on reasonable request.

## Abbreviations

AAU: Addis Ababa University
BSMS: Brighton and Sussex Medical School
CDT-Africa: Centre for innovative Drug development and Therapeutic trials in Africa
CR: Community representative
DM: Decision maker
FGD: Focus Group Discussions
EnDPoINT: Excellence in Disability Prevention Integrated across Neglected Tropical Diseases
HEW: Health Extension Worker
HP: Health Professional
KIIs: Key Informant Interviews
LF: Lymphatic Filariasis
NTD: Neglected Tropical Diseases
TOT: Training of Trainers
TOC: Theory of Change workshops
