# Supplementary material for: A qualitative study on the implementation of a holistic care package for control and management of lymphoedema: experience from a pilot intervention in northern Ethiopia
Source: BMC Health Serv Res. 2021 Oct 8;21:1065. doi: 10.1186/s12913-021-07088-7 (PMC8501530; doi:10.1186/s12913-021-07088-7)
Supplement: Supplementary file 1 — Additional file 1. Topic guide questions english version. [file 12913_2021_7088_MOESM1_ESM.docx]

**Topic guide questions english version**

Greetings! Thank you for agreeing to speak to us. As we discussed previously, this interview is part of our effort to improve care for people affected by podoconiosis, LF and leprosy who have limb care and psycho social care needs.

**Part of our plan is to :**

a.Provide an integrated morbidity management and disability prevention services for people affected by LF, podoconiosis and leprosy

b.Provide a holistic care for these patiens (both physical and psych-social)

c.Support integration of the above services into the routine care, particularly the primary care system.

I would just like to learn from you about the need you see in this area and what integrated and holistic care should look like for these people. There are no right or wrong answers. I value your perspectives.

Do you have any questions that you want to ask me beforehand?

Thank you in advance for your assistance!

1. Let’s start by you telling me a little bit about your experience regarding people affected by podoconiosis, LF and leprosy with limb care and psychosocial care needs and your involvement with them.
2. What do you understand by integrated care?
3. What do you think about the importance and potential impact of integrated care for patients with podoconiosis, LF, and leprosy? Probe for possible gains/disadvantages of integration.
4. • How important and acceptable do you think it is to integrate psychosocial care aspects into the care package?
5. What do you think are the views of policy makers (Health bureau, office and facility manager)? Do you think that they believe that developing an integrated care package as an important, good and acceptable idea?
6. What do you think are the views of providers? Do you think that they view it is an important and acceptable idea?
7. What do you think are the views of patients themselves? Do you think that they view it as an important and acceptable idea?
8. What do you think are the views of caregivers (individuals who care for people with podo, LF or leprosy) Do you think that they view it as an important and acceptable idea?
9. What are the possible barriers for implementing the proposed integrated care package? What would be the strategies to overcome these obstacles? Probe for possible challenges and solutions in relation to policy, human resources, supplies, physical infrastructure, budget, feasibility and sustainability issues (how much external support is needed to make it work e.g. EnDPoINT).
10. Do you think an integrated care package would improve the care of patients? Improve the skills of providers?
11. What do you think are the best ways of stigma mitigation?
12. Would you recommend the intervention to similar patients in other places?
13. Any additional information you would like to add.

**Group Discussion Guide**

**Group Discussion Facilitation Protocol**

1. **Introduction /Warm-up**
   - Thank participants for their willingness to participate in this discussion.
   - Explain the purpose of the group discussion
   - Introduce yourself
   - Explain the role of the facilitator and note takers
   - Let participants introduce themselves
   - Introduce the topic of discussion
2. **Instruction**
   - Assure all participants that personal data will be kept confidential.
   - Make clear what is expected of participants
   - Make clear the time length of discussion. Make sure that the discussion lasts anywhere between 45 minutes and 1 hour.
   - Make sure that each participant in the group fills out the group discussion Participant Profiling Form.
   - Ask for permission to take notes.
   - Keep eye contact with the participants, make sure you shift your attention among all participants and make sure you include everyone.
   - Maintain eye contact with the individual with a disability even if they are blind or are using an interpreter.
   - Do not make any assumptions about limitations.
   - Set ground rules for the group with the participants: Consider the following rules:
   - Respect for different views, no wrong answers, one person speak at a time, everyone has the right to speak without being interrupted, raise your hands and get a signal from the facilitator before you talk, keep your answers short and precise to allow others to participate, switch off/silence your cellphones
   - Do NOT promise what you cannot deliver.
3. **Closing and Post Discussion Activities**
   1. We have had a very good discussion. Summarize the ideas which emerged from the focus group, noting where there was consensus and where there was not consensus
   2. Is there anything anyone would like to add before we close? Probe: go around the group, giving each participant a chance to respond
   3. Thank everyone for their time and input
   4. Make sure to write the group discussion report immediately after the discussion.
